# On-Chip Photonic Detection Techniques for Non-Invasive In Situ Characterizations at the Microfluidic Scale

**DOI:** 10.3390/s24051529

**Published:** 2024-02-27

**Authors:** Tamar Kurdadze, Fabrice Lamadie, Karen A. Nehme, Sébastien Teychené, Béatrice Biscans, Isaac Rodriguez-Ruiz

**Affiliations:** 1 CEA, DES, ISEC, DMRC, Univ Montpellier, 30207 Bagnols-sur-Ceze, Marcoule, France; tamar.kurdadze@cea.fr (T.K.); fabrice.lamadie@cea.fr (F.L.); 2 Laboratoire de Génie Chimique, CNRS, UMR 5503, 4 Allée Emile Monso, 31432 Toulouse, Francesebastien.teychene@toulouse-inp.fr (S.T.); beatrice.biscans@toulouse-inp.fr (B.B.)

**Keywords:** microfluidics, lab-on-a-chip, photonic detection, spectrometry, analytical chemistry, sensors

## Abstract

Microfluidics has emerged as a robust technology for diverse applications, ranging from bio-medical diagnostics to chemical analysis. Among the different characterization techniques that can be used to analyze samples at the microfluidic scale, the coupling of photonic detection techniques and on-chip configurations is particularly advantageous due to its non-invasive nature, which permits sensitive, real-time, high throughput, and rapid analyses, taking advantage of the microfluidic special environments and reduced sample volumes. Putting a special emphasis on integrated detection schemes, this review article explores the most relevant advances in the on-chip implementation of UV–vis, near-infrared, terahertz, and X-ray-based techniques for different characterizations, ranging from punctual spectroscopic or scattering-based measurements to different types of mapping/imaging. The principles of the techniques and their interest are discussed through their application to different systems.

## 1. Introduction

Microfluidics is an interdisciplinary field that involves the manipulation of small volumes of fluids, typically in the microliter to picoliter range. It has evolved from the miniaturization of chemical processes in the 1970s to the development of “lab-on-a-chip” technology and the preceding concept of micro total analysis systems (µ-TAS) in the 1990s [1], enabling nowadays complete analytical laboratories on a single microchip [2,3,4,5,6]. Well beyond the drastic reduction of sample volume, reagent consumption, and waste, this miniaturization provides important advantages for the improvement of the analytical performance, such as an increase of sensitivity, a reduction of analysis time, and the possibility of multiple parallelization without an increase of system complexity and environmental footprint.

Significant milestones of microfluidics progress include the integration of DNA analysis, advancements in chemical synthesis on microchips, and the emergence of “organ-on-a-chip” systems for realistic drug testing. Recent years have seen the rise of soft and flexible microfluidic devices [7], while ongoing research explores technologies like optofluidics [8,9,10], acoustofluidics [11,12], and the combined use of artificial intelligence for enhanced control and optimization [13,14,15]. In line with these advancements, analytical techniques have had to adapt to measurements at these scales, as well as to optimally interface or integrate into microfluidic devices. Therefore, various detection techniques have been adapted to address this issue, such as electrochemistry [16,17], capillary electrophoresis [18,19], mass spectrometry [20,21,22,23], nuclear magnetic resonance [24,25] spectroscopy, and optical detection [26,27,28,29,30,31]. Photonic detection offers particular advantages with respect to other techniques. While the use of passive detection is circumscribed to samples presenting emission properties, active photonic detection schemes, in which samples are subject to interacting with a probing light beam, can be generally considered as non-invasive (i.e., they do not affect sample state), hence becoming of particular interest when they are applied to in operando studies. Additionally, they commonly offer a faster response time than electronic-based techniques while preserving a high sensitivity, which allows for high throughput and rapid analysis, and they are not susceptible to electromagnetic interference [32]. Moreover, photonic techniques allow for multiplexed detection of multiple parameters, valuable in complex sensing applications. In this sense, although on-chip optical detection techniques have proven their value in several applications, such as in the development of point-of-care diagnostics [32], and miniaturized/portable equipment based on classical microscopy and many UV–vis–NIR techniques are commercially available, in other cases the high price of optical components and the requirement of particular bulky devices for technique implementation have limited their practicality in the development of “truly portable” tools for field applications. At the same time, non-optical detection schemes based on electronics (such as electrical, magnetic, and acoustic detection-based devices, which have been the subject of a recent review [33]), while they can be cost-effective and more easily integrated taking advantage of microelectronics developments, can also require the coupling of side and frequently bulky materials and equipment for detection [33].

Putting a special emphasis on the on-chip integration of photonic detection schemes and pointing to new directions toward systems miniaturization, this review article is particularly dedicated to photonic detection techniques, which are extensively used as characterization tools at the microfluidic scale. Without the intention of being a systematic review, and according to the view of the authors, this work aims to comprehensively gather the most relevant and recent advances in the on-chip implementation of UV–vis, near infrared, terahertz, and X-ray-based techniques. Indeed, an overview of the related literature available on the Web of Science Core Collection allows observing that microfluidics advances are seducing a very multidisciplinary scientific community, which, relying on these detection techniques, is adopting microfluidic approaches as a comprehension or analytical tool for very vast and diverse applications. Moreover, the use of these tools has shown continuously increasing interest over the last three decades. Table 1 presents a closer look at the recent (post-2010) literature on the subject. The different photonic detection techniques are classified into three main groups (optical imaging-, spectroscopy-, and scattering-based methods), which will also be used hereafter, and categorized into three main scientific fields of application: chemistry, materials science, and biology. Before entering into the matter, it is worth analyzing this partial picture of the literature, which is representative of the current trends in the implementation of microfluidics to diverse studies of interest, and their coupling to different photonic characterization techniques. It can be observed that the applications in chemistry are leading the rank, followed by material sciences and biological sciences. At the same time, it is worth noting the important weight of optical imaging as the most widespread characterization technique coupled to the microfluidic scale, followed by the ensemble of spectroscopic detection techniques. As it will be discussed hereafter, the observed decreasing weight of the different techniques goes in line with their increasing complexity and difficulty of implementation at the microfluidic scale, which is a trend followed in every scientific field of application. In this sense, the different detection techniques that are presented here will be equally discussed considering their degree of complexity, ranging from punctual spectroscopic- or scattering-based measurements to different types of mapping/imaging, along with their particular interest and limitations. Additionally, for each of the introduced techniques, their fundamental principles are also discussed for the sake of comprehensiveness and to reach a wider audience within the vast diversity of research interests revolving around microfluidics. To this end, a selection of relevant setup configurations and applications is presented.

## 2. Optical Imaging Detection Techniques

Optical imaging detection techniques are the most versatile and, hence, most widely utilized methods in microfluidics. Optical imaging encompasses various approaches, such as bright-field, dark-field, polarized, phase contrast microscopy, and fluorescence-based imaging techniques (including classical fluorescence, confocal, and light-sheet microscopy). Among these, the bright-field and fluorescence-based imaging are the most widely employed methods. The dark-field, phase-contrast, and polarized microscopies will not be discussed herein, thus we invite readers to explore these techniques in the following review articles [34,35,36]. Bright-field imaging is commonly used for high-throughput observation and general sample characterization, i.e., droplets and their manipulations, while fluorescence-based imaging techniques are employed to visualize and study more quantitatively dynamic processes from cellular structures to analyzing genetic material, detecting biomarkers, probing protein behavior, and monitoring chemical processes.

### 2.1. Bright-Field Imaging

Bright-field imaging is probably the most fundamental and widespread microscopic imaging technology. It captures the topographic information of the observed objects when illuminated by a cone-shaped bright beam passing through a condenser and absorbed by the sample, creating a contrast between light and a bright background [27]. This technique is simple and easy to use, is cost-effective, and allows real-time observations [37,38]. Moreover, coupled with high-speed camera imaging, it allows researchers to obtain time-resolved information down to the sub-millisecond level [39]. It is also one of the easiest techniques to couple with a microfluidic device, as most of the materials used in their fabrication are transparent, allowing for straightforward optical imaging. Based on this technique, Heubner et al. [40] developed a water-in-oil microdroplet system to explore the ultrafast kinetics of a chromogenic reaction between Fe^3+^ and CSN^−^ with sub-millisecond (0.5 ms) and sub-micron (0.5 µm/pixel) time and spatial resolution, respectively. To monitor droplet formation, size, and position, Yang and coworkers employed bright-field imaging coupled to a high-speed camera with a detection speed of 3 kHz [41]. Bright-field microscopy has been employed coupled to microfluidics for the screening of protein crystallization conditions [42,43] or for microparticle synthesis [44,45]. With the addition of dye or colored tracers into droplets, the mixing efficiency can also be observed and characterized using high-speed cameras. For instance, Ismagilov and co-workers systematically investigated the mixing phenomena in droplets in both straight [46] and winding microchannels [47]. In the straight microchannel, mixing is driven by recirculating flow, whereas in the winding microchannel, chaotic mixing occurs. Bright-field microscopy can also be used to detect phenomena such as coagulation [48] and agglutination [49]; however, this technique has limitations in scenarios involving low contrast and transparent objects. Its resolution, as in any other classical optical microscopy technique, is constrained by diffraction limit (around 200 nm considering a numerical aperture of 1.5). Moreover, it cannot provide any three-dimensional information about objects. Even though colorimetry has shown to be a quantitative approach for droplets system characterization in bright-field microscopy [50], its sensitivity is limited by the short optical path length of droplets [45].

### 2.2. Fluorescence-Based Imaging

In biological applications, the accurate measurement/detection of compounds at low concentrations is of critical importance. Fluorescence microscopy is a straightforward and effective solution to meet this requirement. The fluorescence principle can be summarized as follows. Following the passage through a medium containing fluorescent probes, the light is absorbed by the probes, prompting electrons to transition from the ground electronic state to the excited state. Subsequently, these electrons release energy by emitting light. The emitted photons are of lower energy (i.e., frequencies) compared to the absorbed photons. Fluorescence-based detections rely on capturing and analyzing this emitted light. The most commonly used fluorescence detection technique is fluorescence imaging. The quantification of analyte concentrations is one of its features. Its sensitivity can be enhanced if extremely sensitive cameras and suitable filter sets are used, allowing ultra-high throughput sample detection. In contrast, in the case of continuous long-term exposure to excitation light, photobleaching and phototoxicity of the probes/samples can occur. Moreover, the choice of materials for microfluidic fabrication and sampling should be made carefully, as many polymeric materials display autofluorescence that can interfere with on-chip optical measurements, leading to high background noise and suboptimal limits of detection in fluorescence imaging and in fluorescence spectroscopy [51]. Nevertheless, a primary limitation of fluorescence-based techniques lies in their dependence on labeling or derivation [52].

#### 2.2.1. Epifluorescence Microscopy

Epifluorescence (also simply named fluorescence) microscopy is highly sensitive and the most classical and widely used technique for detection in microfluidics [34,52,53,54,55]. In a classical configuration setup, an excitation light is directed onto the sample through a dichroic mirror that reflects the shorter wavelength excitation light towards the sample, while allowing the longer wavelength emitted fluorescence to pass through. Epifluorescence microscopy has been actively used for microstructure imaging analysis, including the on-chip quantitative assessment of actin filament assembly dynamics [56] or the creation of solvent–assisted bilayers in microfluidics [57], as they provide fast acquisitions, enabling real-time imaging. Torres-Simon et al., developed an epifluorescence-inverted microscope tailored for multiplex bacterial antibiotic experiments, enhancing bacteria imaging in microdevices. The setup included a blue LED light source, achromatic doublet lenses, and an electrically tunable lens for better focus. The designed cost-effective system accurately detected 100 nm fluorescent beads, achieving the resolution required for single-cell analysis of bacteria, which was also demonstrated by using green fluorescent protein (eGFP)-transfected human fibroblasts as a model of study [58]. Wink and co-workers employed a combination of in-line chip mass spectrometry and on-chip epifluorescence characterization to track the bioactive metabolites produced by actinobacteria incubated in microdroplets [59]. Moreover, on-chip integrated classical fluorescence imaging has been applied for the high-throughput measurement of β-galactosidase activity [60] and for single-nucleotide polymorphism genotyping in microdroplets [61]. However, the epifluorescence microscope has low axial resolution (a few micrometers) and a limited depth of field (a few hundred micrometers), which can make it difficult to image samples with three-dimensional structures.

#### 2.2.2. Confocal Microscopy

In confocal microscopes, the emitted fluorescence is directed through a pinhole with the help of a focusing lens, ensuring that only fluorescence emitted at the focal point reaches the detector. Compared to epifluorescence microscopy, the axial resolution of a confocal microscope is notably enhanced, allowing 3D image reconstruction (as described hereafter); however, it requires a slower acquisition time because the sample to be examined has to be scanned. As with other imaging techniques, coupling to microfluidics can be straightforward and requires no special optical elements. In this regard, Roy et al., used confocal microscopy to visualize the three-dimensional conformation of DNA molecules in a pressure-driven flow inside microchannels (≈70 µm), while epifluorescence microscopy was used for studying the DNA molecules’ 2D dynamics [62]. Schulze Greiving et al., paired confocal microscopy with electrophysiological measurements to characterize the thickness (by coupled surface area and capacitance measurements) and fluidity of the lipid bilayers and to study their impact on cellular ion channel function. For that purpose, fluidity was assessed using fluorescence recovery after photobleaching (FRAP) with a fluorescently-tagged phospholipid (NBD-PE) [63]. Liu and co-workers also took advantage of fluorescence confocal microscopy to characterize the physical and spatial features of microstructures in microporous media [64]. Despite the advantages of confocal microscopy, some drawbacks and limitations must be mentioned, especially when used for long-term exposure observation of biological samples. The confocal microscope excites the fluorophores above and below the focal plane, increasing the possibility of photobleaching. Moreover, some organic molecules in biological samples can be decomposed by long-term laser point-by-point scanning, causing phototoxicity. These drawbacks can be addressed using light sheet fluorescence microscopy (discussed hereafter), which provides high spatio-temporal resolution while measuring samples within the dimension from submicron to centimeters in size [65].

#### 2.2.3. Light-Sheet Fluorescence Microscopy

**Figure 1:**
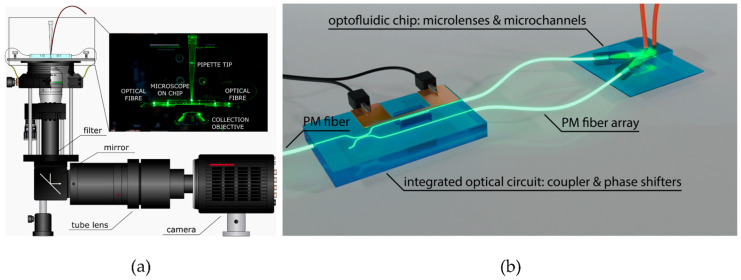
(**a**) Scheme of the custom-made microscope used for imaging of Drosophila embryos. Reproduced from [67], from *Journal of BIOphotonics* published by Wiley 2020. (**b**) Schematic design of SLS-IFC platform: a PM fiber is coupled to an integrated optical circuit designed to symmetrically split the beam and introduce an on-demand phase shift between the two outputs. The collected signal, transmitted through PM fibers, reaches an optofluidic chip, where microlenses generate two overlapping light sheets within a microfluidic channel, forming patterned illumination light. Reproduced from [68], from the journal *Lab on a Chip* published by the Royal Society of Chemistry 2024.

Light-sheet fluorescence microscopy achieves selective specimen illumination using a cylindrical lens, which focuses a thin light sheet onto the sample. This thin light sheet selectively excites the fluorophores in the focal plane, while minimizing the exposure above and below. The sample is then imaged perpendicular to the light sheet, providing clear and high-contrast imaging [27]. Due to these advantages, light sheet microscopy is an interesting tool for dynamic studies in microfluidics. In this regard, Jiang et al., developed a microfluidic device coupled with light sheet microscopy for high throughput sample preparation and quantitative analysis. Screening of 4D (space–time) at a high speed (500 fps) of droplets/plugs with a throughput of 30 droplets per minute was achieved, and fluorescent micro-particles encapsulated in the droplets were quantified [66]. Memeo et al., fabricated a light-sheet microscope on a chip for automatic imaging of Drosophila embryos. The device integrated a cylindrical lens on-chip to focus the light from an optical fiber in a singular direction, creating a light sheet that intersected the microfluidic channel. Integrated waveguides were used to precisely couple and align two counter-propagating light sheets to uniformly illuminate the entire sample (Figure 1a) [67]. Paiè and coworkers introduced an innovative automated platform capable of performing structured light sheet imaging flow cytometry (SLS-IFC), which offers exceptional capabilities in 3D imaging of individual cells flowing through microfluidic channels, improving the spatial resolution of conventional fluorescence microscopy. The platform could generate light sheets of blue and green light of 488 nm and 561 nm, respectively. Several interference patterns with different spatial frequencies could be created by varying the angle between these light sheets. This feature enhanced the system’s versatility, enabling the simultaneous study of several cellular components. The integrated optofluidic platform comprises a multi-wavelength directional coupler, a thermo-optic phase shifter, and cylindrical lenses designed to generate and shift a patterned light sheet within a microchannel. It also involved two distinct glass components connected by polarization-maintaining (PM) optical fibers. The first optical chip incorporated a directional coupler and a thermal phase shifter, facilitating rapid and on-demand phase shifting between the two arms of the coupler (Figure 1b). The second optofluidic device, consisting of cylindrical microlenses and a microfluidic channel, was conceived for imaging detection. Each lens collected light from the corresponding optical fiber, generating a light sheet that overlapped precisely in the microfluidic channel where the sample flowed. In this approach, the cells flowed through the illumination plane, enabling the automatic imaging of the fluorescence signals and performing dual-color SLS-IFC in real-time and motion [68]. The system was able to detect details below the diffraction limit, such as vesicles within the volume of HeLa cells, as well as nanoparticles taken up by the cells at different concentrations. The system achieved remarkable acquisition speed, capturing an entire stack of 30 slices of a single cell in approximately 1.5 s, with a capacity of up to 40 cells per minute.

## 3. Spectroscopy-Based Detection Techniques

Spectroscopy-based detection techniques allow for retrieving information about analyte structure, composition, and properties by studying its interaction with electro-magnetic radiation. As widely used techniques for in situ characterizations at the microfluidic scale, the coupling of vibrational, UV–vis, and X-ray absorption spectroscopies will be discussed in this section. Vibrational techniques are mainly, but not exclusively used for analyte determination, as the identification of unique molecular fingerprints can provide high sensitivity and selectivity for compound detection. When analytes present specific light absorption in the UV–vis range, UV–vis spectroscopy is the method of choice for quantitative analysis, mainly for the well-known linear response for analyte concentration changes within a specific range. Meanwhile, X-ray absorption spectroscopy, although being a technique of more restricted use (due to the high costs of equipment and the limited access to synchrotron radiation facilities), is particularly useful for revealing the structures on an atomic level (using XAFS, X-ray absorption fine-structure analysis) and to study analyte speciation, as it provides a selective and sensitive analysis of elements and its oxidation state (through XANES, X-ray absorption near edge structure analysis).

### 3.1. Vibrational Techniques

#### 3.1.1. Raman Spectroscopy

Raman spectroscopy is a widely used optical detection technique that derives the chemical structure information from the inelastic scattering of molecular bonds upon laser illumination. It relies on the interaction between photons from a laser source and molecules in a sample. When these photons scatter off the molecules, a small portion undergoes inelastic scattering (Raman scattering), resulting in energy changes that correspond to molecular vibrations or rotations. By analyzing the frequency shifts (Stokes or anti-Stokes) in the scattered light, Raman spectroscopy unveils a unique fingerprint of the sample’s chemical composition and structure, enabling precise identification and characterization of materials without the need for extensive sample preparation. Analytes can be identified through Raman shift and quantified by assessing the corresponding peak intensities. It has multiple attractive characteristics, such as label-free and non-invasive detection, a rapid response, and the possibility of performing online analyses [69,70,71]. Employing Raman spectroscopy on a chip, Lines et al., proposed a microfluidic system to miniaturize the analyses of hazardous radioactive samples related to the plutonium–uranium reduction–extraction process (PUREX). They first demonstrated the accurate quantification of species in a solution (HNO_3_ and NaNO_3_) using chemometric analysis, despite the presence of overlapping or confounding spectroscopic bands [72]. Later, this team proposed a similar setup to study biphasic extractions involved in the PUREX process. HNO_3_, as the aqueous phase, and 30% (*v*/*v*) tributyl phosphate in n-dodecane, as the organic phase, were used, simulating the PUREX conditions. The HNO_3_ concentrations were monitored in both the aqueous and organic phases throughout the extraction process. The extraction kinetics results were compared with macroscale kinetics, confirming the validity of the proposed miniaturized approach [73]. Wang et al., introduced an integrated Raman-activated droplet sorting (RADS) microfluidic system for the functional screening of live cells, using an industrial microalga as a model system. The Raman spectra of individual cells were obtained prior to encapsulation in water droplets in oil. Successful sorting was demonstrated with 98.3% accuracy, an enrichment ratio of eightfold, and a throughput of ~260 cells/min [74]. Often, conventional Raman spectroscopic and microscopic systems face challenges in collecting data from microfluidic chips due to substrate background signals. To address this issue, Kim et al., developed a Raman spectroscopy setup coupled with a PDMS droplet microfluidic device, minimizing the PDMS Raman spectral background using a confocal pinhole and an inverse device orientation, and achieving high-throughput single-cell resolution for characterization of microalgal lipid production over time within the droplets [75]. The same issue was tackled by Ashok and co-workers by integrating fiber probes on-chip. An excitation fiber was directly inserted into a PDMS microfluidic channel, while a collection fiber was equally positioned perpendicularly, eliminating substrate interference in the collected spectra [76]. Moreover, pre-aligned mirrors, lenses, and fiber optic guides have been integrated into a microfluidic chip, demonstrating 70% unidirectional optical throughput and no spectral artifacts in the wavelength of 200 to 800 nm for Raman and fluorescence spectral measurements [77]. Likewise, an ultrafine fiber Raman probe (30 µm) with a high spatial resolution (23 µm) was developed for the non-invasive molecular diagnosis of organs [78]. Detection of low-concentration samples is challenging with conventional Raman spectroscopy due to its limited sensitivity. These limitations are improved by six to ten orders of magnitude by electromagnetic field and chemical enhancement effects generated by nanostructures, provided by the surface-enhanced Raman scattering technique (SERS). With a detection limit at the single-molecule level, SERS is considered as an ultra-sensitive technique [79,80]. Importantly, SERS eliminates any interference from water and does not cause damage to samples, making it suitable for the analysis of biological samples [81,82,83,84,85,86] or cancer diagnostics [87,88,89,90,91]. Equally, microfluidic sensors based on SERS detection have been proposed for very diverse applications, ranging from the diagnosis of Alzheimer’s disease [92] to the rapid analysis of food contaminants [93] or the high-throughput detection of explosives [94] or harmful substances [95]. Li and co-workers developed a novel microfluidic SERS sensor integrated with a side-polished multimode fiber (SPMF). SPMF SERS probes were fabricated by depositing Au nanorods on a planar polished fiber surface. SPMF provided a large planar surface for light–matter interactions and a large SERS interaction area, increasing the collision probability between the tested molecules and the SPMF SERS probe, and improving the SERS sensitivity up to 10^−8^ M and the relative standard deviation (RSD) to less than 10% for malachite green (used as a pesticide contaminant model) with fairly short integration times below the second. Subsequently, the system was proposed for its potential application in environmental science and biomedicine. The detection of pesticides (thiram) and antibiotic (levofloxacin) residues in tap water was demonstrated down to 10^−9^ M and 10^−6^ M concentrations, respectively [96].

#### 3.1.2. Fourier-Transform Infrared Spectroscopy

FTIR spectroscopy is based on the detection of vibrational energy levels of specific chemical bonds using a broadband infrared light source, assessing light absorption by specific molecules, and detecting sample composition, concentration, structure, and molecular interactions. FTIR, like other photonic techniques, uses a light source and a sensor. Its specificity comes from using infrared light with wavelengths ranging from 1600 to 25,000 nm that could require the use of special materials like CaF_2_, sapphire, ZnSe, or silicon for chip fabrication, as conventional microfabrication materials like glass are not fully transparent in this kind of spectral range. It is a label-free and due to the low energy levels of infrared photons that cannot damage chemical bonds, it is also considered a non-invasive technique [97]. Despite the growing interest in integrating Fourier-transform infrared spectroscopy (FTIR) in microfluidic systems, it is not yet a widely adopted technique. Herein, some highlights of FTIR spectroscopy on-chip detection together with FTIR spectroscopic on-chip imaging techniques are presented.

Chan et al., proposed FTIR spectroscopic imaging for time-resolved mapping of chemical composition, allowing for the study of segmented flow at a velocity of 2.5 mm/s, with a temporal resolution of 120 ms, using IR spectrometer and a 64 × 64 focal plane array (FPA) detector. These images could potentially be used to visualize fast chemical reactions in multiphase segmented flows [98,99]. Later, the same team studied a model chemical reaction, the neutralization of benzoic acid in decanol with disodium phosphate in water, using the previously mentioned system. The concentration profiles of the reactants and products were imaged and quantified at different times and positions [100]. Landari et al., proposed an FTIR-based pseudo-continuous flow microfluidic device for glucose, fructose, and sucrose detection and quantification with applications in food industry control. The system consisted of three main parts: pumping subsystems, a microscope-FTIR spectrometer, and a microfluidic chip with a heating system. The latter was used to evaporate the solvent, therefore permitting the researchers to identify and quantify sugar types in aqueous solutions. They attained a 4.35% measurement error, which represents a 10-fold improvement with respect to conventional measurements. [101]. Indeed, the main drawback of FTIR spectroscopy is that many solvents, especially water, have strong absorption in IR spectra, limiting the application of the technique in aqueous media and hampering its use for biological applications [102]. In this regard, attenuated total reflection Fourier transform infrared spectroscopy (ATR-FTIR) is an effective approach for studying biological samples, both hydrated and dried, such as cells and fluids [103]. The sample and the ATR element are in full contact, reducing the effective path length of IR light within the sample, thus preventing the absorbance of water bands from saturating the signal received by the detector. When the beam is sent at a critical angle, it can reflect multiple times within the crystal, creating an evanescent wave that extends beyond the ATR element. This evanescent wave loses energy at frequencies identical to the sample’s absorbance [103].

**Figure 2:**
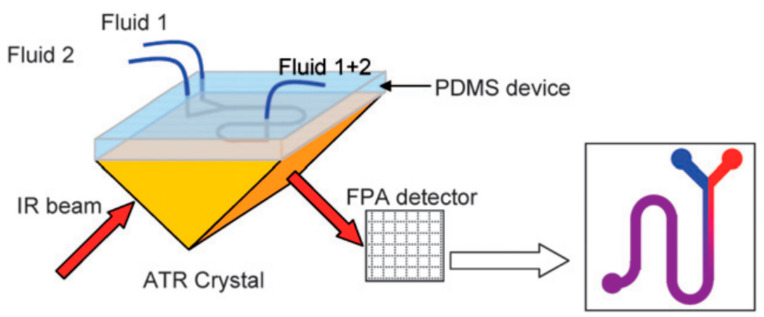
Schematic of ATR-FTIR imaging system integrated with a planar microfluidic chip. Reproduced from [104] with permission from the Royal Society of Chemistry. Copyright 2009; permission conveyed through Copyright Clearance Center, Inc.

In this line, Chan et al., developed an inverted prism-shape ATR crystal (see Figure 2) integrating a PDMS microfluidic chip to image the mixing of liquids, demonstrating the potential of this approach for a label-free, high-throughput, and quantitative analysis of various chemical and biological systems [104].

Recently, Jia et al., developed what they called “SpectIR-fluidics” by integrating a multi-groove silicon ATR crystal into a microfluidic device for FTIR spectroscopic measurements. This integration allowed real-time continuous chemical mapping, rapid tracking of dynamic concentrations, and parallel high-sensitivity measurements with low limits of detection, such as 540 nM for D-glucose. The key features of this device include support for world-to-chip connections, compatibility with closed microfluidic channels of arbitrary complexity, and minimal dead volume space, providing flexibility in device design. That makes “SpectIR-fluidics” a powerful tool for lab-on-a-chip biological and chemical applications [105]. Srivastava et al., proposed a custom-designed single-bounce ATR-integrated microfluidic reactor to obtain in situ time-resolved information on chemical reactions using synchrotron IR radiation. As a proof of concept, they characterized the model second-order nucleophilic substitution (S_N_2) reaction of benzyl bromide (BB) and sodium azide (SA), producing benzyl azide (BA) [106].

#### 3.1.3. Terahertz Spectroscopy

Terahertz radiation (THz) is positioned between microwaves and infrared regions in the electromagnetic spectrum (frequencies ranging from 0.1 to 30 THz). It is gaining interest due to its non-ionizing and low-energy nature, which provide resonant excitation frequencies able to interact with the rotational and vibrational modes present in biological samples without causing tissue damage. THz measurements allow real-time measurements [107] with high spectral and spatial resolution and a fast response [108]. However, studying aqueous samples by THz spectroscopy is still challenging due to the high absorption of water in this frequency range, causing an insufficient signal-to-noise ratio. The minimization of sample thickness (i.e., optical path length) down to a few tens of micrometers, thanks to microfluidic dimensions, prevents excessive water absorption, therefore enabling spectroscopic measurements in aqueous media. THz spectroscopy is, however, not yet regularly employed in microfluidics due to the challenges related to instrumentation, signal processing, and material choice. Furthermore, it should be noted that most of the proposed microfluidic chips for THz spectroscopy feature relatively large dimensions for the detection area to improve the signal-to-noise ratio by avoiding absorption of THz waves by the channel walls. In this regard, the choice of material for microfluidic fabrication must be made carefully to achieve low absorption of THz frequencies while achieving accurate and reliable spectral data acquisition [109].

**Figure 3:**
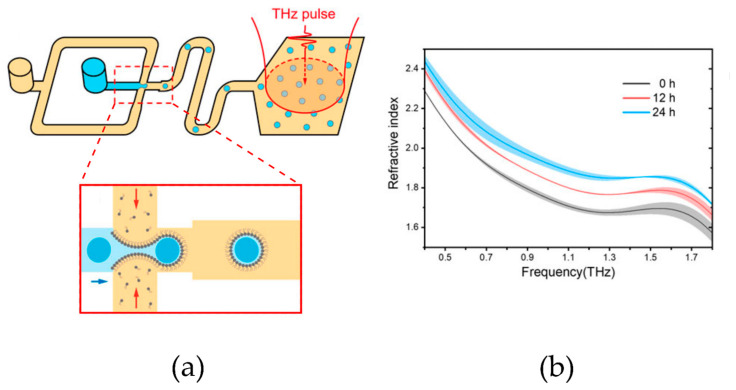
(**a**) Schematic representation of the microfluidic device integrated with automated droplet sampling and THz measurements. (**b**) Refractive index spectra of liver cancer cells after treatment by resveratrol drug for 0, 12, and 24 h. Figure adapted from [115], from the journal *Frontiers in Bioengineering and Biotechnology*, section Nanobiotechnology published by Frontiers 2023.

Terahertz time domain spectroscopy (THz-TDS) is a technique that uses short pulses of terahertz radiation to probe the properties of matter. The generation and detection scheme is sensitive to the effect of the sample on the amplitude and phase of the terahertz radiation. By measuring in the time domain, the technique can provide more information than conventional Fourier transform spectroscopy, which is sensitive only to amplitude. The pulsed nature of the radiation used provides access to quantities such as the complex refractive index of materials by measuring the delay caused by the passage of the sample. Coupled with microfluidic devices, this measurement technique covers a wide range of applications in chemistry and biology [110]. Usually, due to the high absorption of water, THz detection only allows the examination of dried or partially dried samples. However, George et al., coupled microfluidics with a low-power THz-TDS system to measure the absorption coefficient of protein bovine serum albumin in an aqueous solution in the 0.5–2.5 THz frequency range, achieving a detection sensitivity on the picomolar range. The setup consisted of an n-InAs emitter, a cyclo-olefin polymer microfluidic device, and a 1 mm ZnTe electro-optic detector [111]. Baragwanath et al., developed a silicon-based THz-microfluidic cell and tested the sensitivity of the device through a range of experiments involving primary alcohol/water mixtures, commercial whiskeys, and biological molecule–biotin in a solution. The sensitivity of the results were demonstrated while operating with optical path lengths as small as 50 µm, enabling detection quantities in the order of 2 µmol for primary alcohols in a solution and 3 nmol biotin concentration changes in water [112]. Following the same idea, Liu et al., monitored isopropyl alcohol–water (IPA/water) mixtures on-chip, with a fast response of around 10 ns, totally suitable to study ultrafast in situ dynamics of chemical and biological phenomena [108]. THz spectroscopy has equally been employed for cell viability assays. Tang et al., developed a PDMS dielectrophoretic cell-trapping device coupled with THz detection. The system displayed high noise levels due to the high absorptions of aqueous media and chip material itself [113]. To overcome this issue, Yang et al., proposed to use fluorinated oil instead of water, due to its low absorption and lowest cytotoxicity, to monitor human breast cancer cells (MDA-MB-231). However, the system presented other drawbacks, such as the insolubility of the cells in fluorinated oil, which made it difficult to disperse them in the oil media, thus limiting the system application to adherent cells only [114]. In this line, Zhang and co-workers developed a novel microfluidic platform combining on-chip droplet sampling and THz measurements. Cells including bacteria, stem cells, and cancer cells were encapsulated individually in aqueous droplets formed by self-assembled phospholipids, which were dispersed in hexadecane. The droplets containing the cells were analyzed in the detection module containing quartz windows, allowing for high transmission of the THz spectra (Figure 3).

This approach improved the signal-to-noise ratio while preserving cellular viability and allowed for successfully determining the refractive indices of the cells. The high sensitivity and repeatability of the method permitted effective discrimination of cellular states and stress responses, demonstrating its versatility in biomedical applications [115]. THz spectroscopy coupled to microfluidics has also been applied to the characterization of xanthan gum colloids [116] and hydroxygraphene [117].

### 3.2. UV–Vis Techniques

#### 3.2.1. Absorption Spectrometry

**Figure 4:**
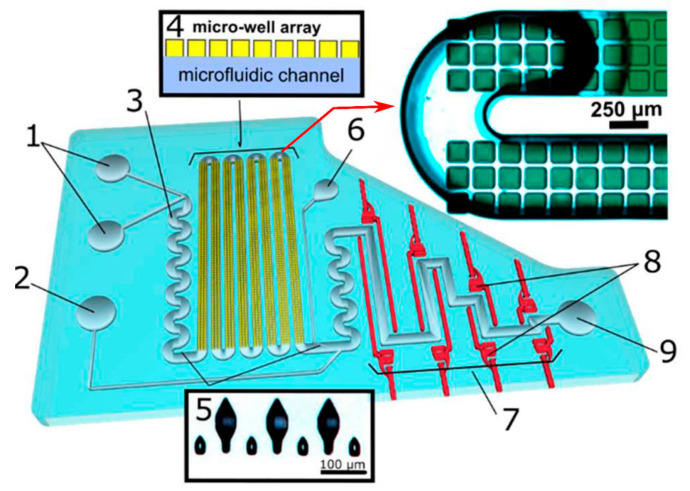
Schematic representation of the OCER platform: 1. inlet ports; 2. additional inlet port; 3. passive zigzag micromixer; 4. serpentine channel for droplet storage (2000 droplets of 2 nL) featuring a cross-sectional view of the solution-storage layout for enzyme crystallization and further cross-linking of the crystals; 5. structures before and after serpentine channel to prevent the dragging of non-fixed crystals/aggregates by injected solutions; 6. Outlet for the crystallization and cross-linking solution to avoid contamination of sensing region; 7. multiple path configuration for the photonic detection system, enabling exploration of a wide concentration range; 8. in red: 2D microlenses with air mirrors along the interrogation channel to prevent cross-talking, self-alignment elements for fiber optics alignment and clamping. Fiber optics are connected to an external light source and spectrometer for on-chip real-time analyses; 9. outlet port for the product solutions. Reprinted with permission from [123]. Copyright 2016 American Chemical Society.

**Figure 5:**
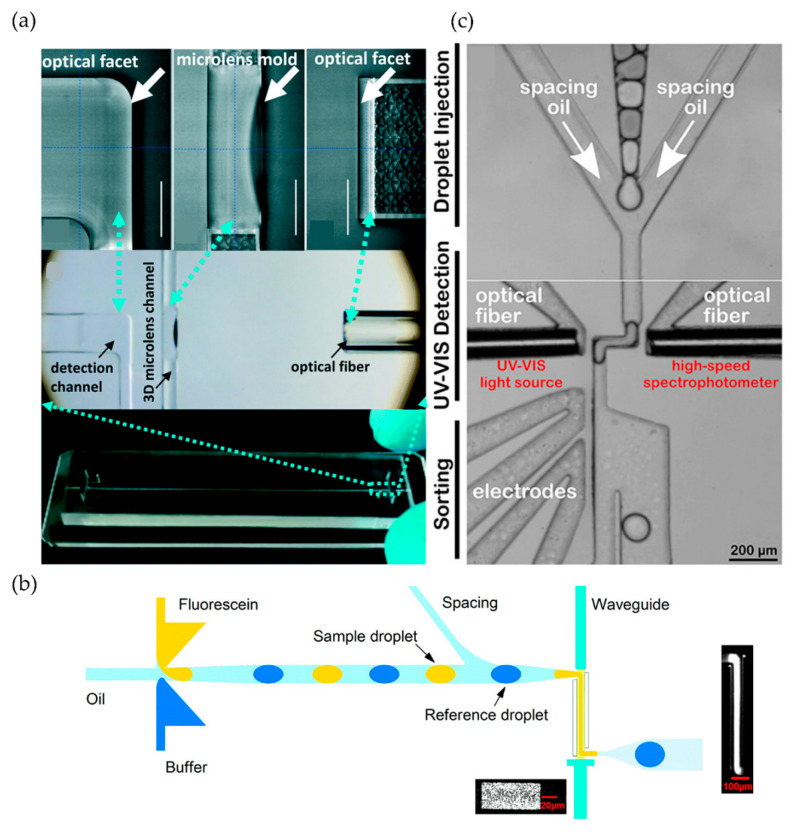
(**a**) Three-dimensional microlens-incorporating microfluidic chip (3D-MIMC) with large optical path length and incorporated optical fiber. Reproduced from [124], from the journal *Lab on a Chip* published by the Royal Society of Chemistry 2020. (**b**) Enlarging the optical path length of the droplet by using droplet stretching for high-sensitivity measurements. Figure adapted from [128], from the journal *Analytical Chemistry* published by the American Chemical Society 2021. Copyright 2017 American Chemical Society. (**c**) UV–vis spectra activated droplet sorter (UVADS) for high-throughput label-free chemical identification and enzyme screening. Adapted with permission from [126]. Copyright 2017 American Chemical Society.

UV–visible absorption spectroscopy is the most widely used optical detection technique in flow-based chemical analysis, as it is universal for almost all organic compounds and enables label-free, real-time quantitative analysis [118]. The principle of this technique relies on the Beer–Lambert law that describes the light attenuation with the propagation inside the medium. Considering a wavelength λ, the absorption is proportional to the optical path length, the concentration of the analyte, and the molar attenuation coefficient of the analyte, which varies as a function of the wavelength. Taking advantage of photonic lab-on-a-chip technology [10] (light coupling and decoupling to the system employing fiber optics and monolithically integrated micro optical elements), on-line on-chip UV–vis detection was proposed by Rodriguez-Ruiz et al., for continuous catalytic enzymatic reactions monitoring, with potential applications for continuous sensing of contaminant detection or for the production of high added value compounds [119]. The main challenges with UV–vis spectrometry are achieving a low limit of detection (which is a limiting factor in the constrained small microfluidic optical path lengths) and covering a large range of concentrations while preserving the linearity range to avoid saturation phenomena. This can be addressed by adjusting the optical path length. In this regard, Rodriguez–Ruiz and co-workers proposed a multiple path photonic lab on a chip (MPHIL) incorporating 2D microlenses for UV–vis spectrometric detection of protein concentration [120] and later, species in solution [121,122]. An analogous approach in terms of a multiple optical path was proposed by Conejero-Muriel and co-workers [123]. They presented an optofluidic CLEC-based (cross-linked enzyme crystal) reactor, OCER, proposed for sensing applications and for the synthesis of high added value products. This sensitive, robust, reusable, and stable platform was specifically designed for in situ crystallization and crystal cross-linking generating the enzymatically active material that can be stored for long periods (months to years, in contrast to weeks for other sensors based on bioenzymatic materials). The integration of micro-optical elements allowed for continuous monitoring of different enzymatic reactions by UV–vis spectrometry. The schematic representation of OCER is given in Figure 4. The chip’s multiple paths configuration allowed for measuring a wide range of p-nitrophenol (p-NP) concentrations (>3 orders of magnitude) ranging from 0.78 µM to 1 mM, demonstrating linearity within high confidence levels (R^2^ = 0.992). Tang et al. [124] introduced a 3D microlens-incorporating microfluidic chip (3D-MIMC) obtained by two-photon stereolithography, featuring an extended detection channel and integrated optical fiber (Figure 5a). The incorporation of a 3D microlens led to a 9- and 4-fold increase in light coupling efficiency and signal-to-noise-ratio, respectively. The sensitivity and the limit of detection (LoD) of the 3D-MIMC assay was improved by one order of magnitude compared to the conventional 96-well plate assay. The abovementioned issue addressing LoDs is a bottleneck, especially for the segmented flow microfluidics. Mao and co-workers developed a droplet-based microfluidic chip integrating an optical fiber-based spectroscopy unit for the measurement of absorption spectra [125]. The absorption detection scheme was addressed by simply facing two optical fibers beside a microfluidic channel; hence, the attained optical path lengths were equivalent to the microchannel dimensions, yielding measurements with limited sensitivity.

In this sense, Yang and co-workers proposed to tackle this issue by stretching the droplet/plug across the channel, (Figure 5b) [126]. A microfluidic channel with a 35 µm × 150 µm cross-section was narrowed down to 35 µm × 26µm wide in the detection region. The droplets could hence be stretched to fill a channel corresponding to an optical path length of 700 µm and 800 µm, which allowed the researchers to respectively obtain the detection limits of concentrations of 406 nM and 276 nM for fluorescein. These values were the first lowest concentration reported for an absorption analysis of single pL volume droplets. On the opposite side, and to characterize highly concentrated solutions, Rodriguez-Ruiz et al., developed a device presenting a gradually shrinking channel, reaching a 150 µm optical path length for the characterization of highly concentrated lysozyme solutions while maintaining absorbance linearity range [127]. Duncombe and co-workers also employed a droplet-stretching design, in this case, for a UV–vis spectrum activated droplet sorter (UVADS) system for high-throughput label-free chemical identification and enzyme screening (Figure 5c). This platform collected full UV–vis spectra at a high frequency and enabled the direct protein quantification down to 10 µM of bovine serum albumin at 280 nm (using a 100 µm optical path length). The extended range of the acquired spectral information could detect unexpected events, such as air bubbles, which would be undetectable at a single wavelength and could distinguish the content of 12,700 droplets in less than a 7 min period [128]. The fast acquisition frequencies, together with the relatively simple detection schemes and technique implementation also make UV–vis techniques one of the favorite candidates for online characterizations in scientific applications related to materials synthesis. Here, the in situ information can be used either for reaction screening [129] or for fine-tuning the production of tailored particles of desired properties. In the same way, Wu et al., proposed a 3D microfluidic chip for the continuous flow synthesis and real-time spectroscopic monitoring of zinc oxide (ZnO) nanostructures. The device consisted of three combined modules, a 3D concentration gradient generator, a mixing unit, and integrated optical fibers for inline monitoring. The spectrometer capable of averaging spectra over 10 ms enabled high precision and rapid on-line monitoring of the concentration changes [130].

#### 3.2.2. Light Extinction Spectrometry (LES)—Turbidimetry

Light extinction spectroscopy and turbidimetry are related techniques. They both measure the decrease in light intensity in a solution; however, they are based on distinct principles. Turbidimetry specifically focuses on the reduction of light intensity caused by the absorption and scattering of particles, providing the overall cloudiness and turbidity of the media. Meanwhile, light extinction spectroscopy involves the measurement of light attenuation across the spectrum of wavelengths, allowing for a more detailed analysis of the interaction between light and particles or absorbing species in a sample, providing information about specific properties of the particles or substances within a sample. The turbidimetry has been employed on lab-on-a-chip devices for rapid, automatic, and sensitive detection of foodborne pathogens, such as viable Salmonella [131], and water contaminant detection [132]. Coliaie et al., developed a continuous-flow microfluidic device with an integrated turbidity sensor, facilitating the in-line measurements of liquid–liquid phase separation boundaries of two ternary systems: β-alanine-water-isopropyl alcohol at room temperature and β-alanine-water-ethanol at 50 °C. The device employs uniform mixing using cyclonic flow in a micromixer and utilizes a turbidity sensor comprising an infrared LED light source and a photodiode. By adjusting the flow rates of the solvent, antisolvent, and solute streams, the device allows for precise detection of binodal points in the liquid–liquid phase diagram, presenting an efficient and high-throughput approach for liquid–liquid phase separation detection in drug development and biotechnology applications [133].

**Figure 6:**
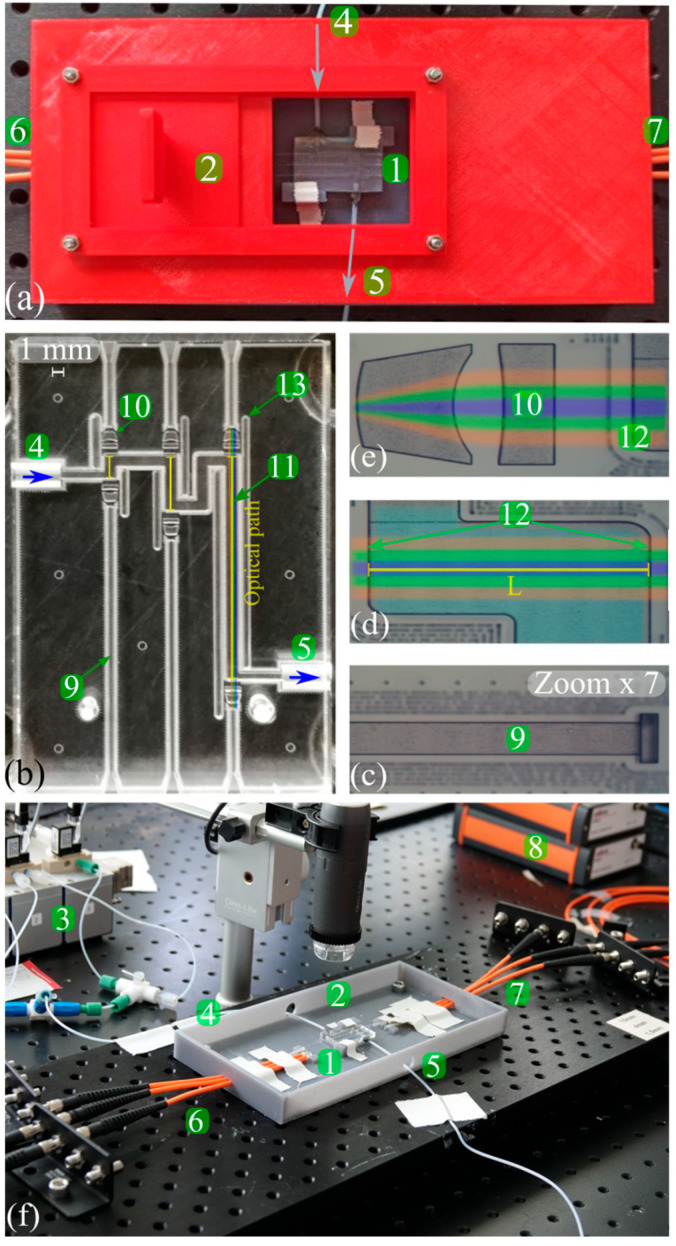
Experimental setup for the photonic lab-on-a-chip with three optical path lengths used for light extinction spectrometry measurements. (**a**) global view of the device in a 3D-Printed hausing; (**b**) detailed view of the chip. (1) Glass chip in its (2) housing (hatch open); (3) computer-controlled syringe pumps (×2) feeding the LoC via its (4) inlet and (5) outlet; (6,7) optical fiber connections; (8) CCD spectrometers. (**c**–**e**) Expanded views of (9) the self-alignment channels (×6) for optical fibers; (10) two-dimensional collimating microlens assemblies; (11) optical channels with path lengths L = 1.0, 3.5, and 10 mm, width 650 µm, and depth 250 µm; (12) channels’ end with parallel optical-grade windows; (13) air channels to avoid probing channels cross-talking during simultaneous acquisition. (**f**) Setup with (2) LoC housing cover removed. Reproduced from [136], from the journal *Optics Express* published by Optica Publishing group 2022.

Light extinction spectroscopy, as a more comprehensive technique, has been primarily used to characterize spherical particles, but also to characterize crystals or aggregates with a small fractal dimension [134]. Moreover, particle size distributions (PSDs) and concentrations in plasmas, aerosols, and colloidal suspensions can be obtained by LES [135]. In principle, LES uses the extinction of a collimated broadband light beam to recover the particle size distribution (PSD) and the absolute concentrations in number or in volume. A novel lab-on-a-chip approach utilizing light extinction spectrometry for measuring particle size distributions and volume concentrations in optically dilute colloidal suspensions has been introduced by Onofri et al. [136]. The photonic lab-on-a-chip platform containing multiple path lengths (Figure 6) offered absolute particle concentration measurements for both stationary and dynamic suspensions, for particle sizes ranging from 30 nm to 0.5 µm and concentrations from 1 to 1000 ppm. Unlike conventional dynamic light scattering (DLS), this approach allowed straightforward implementation in continuous flow and a simultaneous analysis of suspensions with concentration changes spanning up to three orders of magnitude, using minute amounts of the sample.

#### 3.2.3. Photothermal Spectroscopy

**Figure 7:**
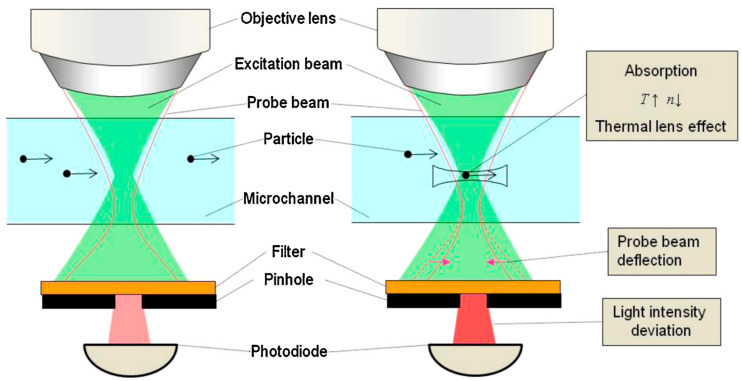
The schematic representation of nanoparticle detection by thermal lens microscope (TLM). No thermal lens effect occurs when there are no nanoparticles present (left). The probe beam is deflected due to the thermal lens effect, causing deviation in probe beam intensity after the pinhole (right). Reprinted from [138]. Copyright (2016), with permission from Elsevier.

In contrast to the techniques based on light attenuation, photothermal spectroscopies offer signals that are weakly dependent on the optical path length, making them highly compatible with microfluidic dimensions, while providing detection limits comparable to fluorescence. The main drawback of these techniques is related to the high cost of the required equipment [52]. Photothermal methods utilizing the thermal lens (TL) effect are valuable tools for sensitive detection of non-fluorescent, non-labeled molecules within microchannels. When exposed to a focused laser beam, mostly in the infrared or near-infrared range, the absorbed energy is converted into heat by non-radiative relaxation following a profile corresponding to the beam’s focus. As liquids typically exhibit a negative temperature coefficient of the refractive index (d*n*/d*T*), the central region of the excitation beam experiences a lower refractive index compared to the surrounding solution, inducing a concave lens effect, known as the thermal lens effect [137]. An excitation beam and a probe beam are focused in the same objective lens, so the small shift of the two laser focal points leads to the refraction of the probe beam due to the thermal lens effect (Figure 7). Theoretically, the ideal magnitude of this shift corresponds to the confocal length of the probe beam. In this regard, Yamaoka et al., employed thermal lens microscopy (TLM) for on-chip detection of 500 nm red polystyrene particles with 85 ± 6% detection efficiency (Figure 7) [138].

Liu and co-workers developed a microfluidic TLM device for flow-injection analysis for high-throughput and sensitive analysis of sub-µL samples, demonstrating a LOD of 0.6 ng/mL for Cr(vi) in high-speed flows (10 cm/s) [139]. Based on the TL effect, Maceiczyk et al., introduced a differential detection photothermal interferometry (DDPI) technique for high sensitivity and ultra-fast (10 kHz) picoliter and femtoliter droplet single-point absorbance measurements. Droplets containing 1.4 µM of erythrosine B were detected at 1 kHz frequency. Furthermore, this technique was employed for enzyme kinetics analysis of β-galactosidase and the evaluation of metabolic activity of HL-60 cells at the single-cell level [140]. Based on the TL effect, Zhou et al., introduced a novel miniaturized detector consisting of a portable photothermal PT-chip coupled with nanomaterial-mediated photothermal effect, which allowed for the visual quantitative detection of biomarkers. They demonstrated a LoD of 2.1 ng/mL for prostate-specific antigen (PSA) in human serum, meeting clinical diagnostic requirements. The photothermal effect was facilitated by iron oxide (Fe_3_O_4_) nanoparticles. The PT-chip utilized a sandwich-type ELISA (enzyme-linked immunosorbent assay) device, where the captured antibody is immobilized on the chip microwells’ surface specifically bound to PSA, while Fe_3_O_4_ is also bound PSA. The Fe_3_O_4_ nanoparticles were converted into a strong near infrared photothermal probe through a simple complexation reaction and exploited to generate heat under laser irradiation. The heat increased the vapor pressure inside the microwell of the PT-chip, driving colored sample solutions to a connected microchannel. The distance traveled by the solution in the microchannel was correlated with the original concentration of the targeted PSA [141]. Abraham et al., developed a microfluidic resonator for the determination of thermal properties of liquid analytes by photothermal modulation. The system exploited a laser diode for the effective heating of the liquid that induced thermal stress on the walls, contributing to the rise of the resonance frequency of the microfluidic resonator. The resonance frequency shift of the resonator provides real-time information about the thermo-mechanical characterization of liquids, such as volumetric expansion and heat capacity. This allowed the characterization of the properties of eight different liquids at sub-nanoliter volumes [142].

#### 3.2.4. Fluorescence-Based Spectroscopy

**Figure 8:**
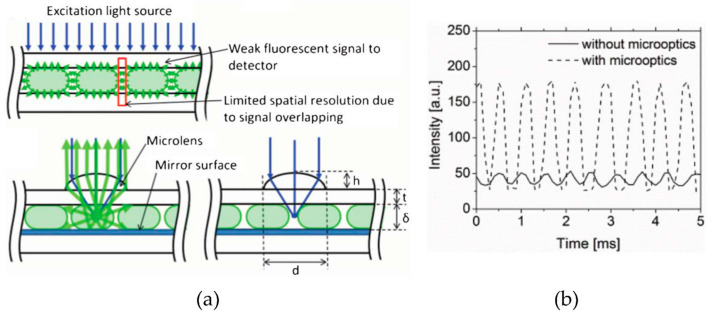
(**a**) Schematic representation of on-chip fluorescence detection in droplet microfluidics with an integrated microlens and a metallic mirror. (**b**) Comparison of fluorescence intensity obtained by the conventional device and the chip integrated with micro-optics. Figure adapted from [144], from the journal *Lab on a Chip* published by the Royal Society of Chemistry 2013.

Fluorescence spectroscopy is a widely implemented optical detection technique in microfluidics due to its low background noise, high sensitivity, and fast response in time. Fluorescence spectroscopy setups are similar to those used for UV–vis spectroscopy in terms of equipment. Light can be delivered to and collected on the microfluidic chip either using optical fibers or microscope setups. However, the light sources are often lasers, and the sensors are equipped with colored filters. Unlike absorption spectroscopy, it is not limited by optical path lengths, as it is based on fluorescent light emission. In this regard, Guo and coworkers developed an optofluidic chip for ultra-high-throughput, real-time fluorescence characterization of 2000 droplets per second with a 20 nM limit of detection [143]. The device had integrated fiber optics. An input fiber for delivering excitation light and a detection fiber located 27° from the input were implemented to maximize the fluorescence detection efficiency and to avoid direct collection of excitation light. This device was used to detect DNA contents in droplets and to analyze single nucleotide polymorphism (SNP) for the ovarian cancer gene. Lim and co-workers introduced a micro-optical lens array for high-throughput fluorescence detection of droplets [144]. The microfluidic device, containing a micro-lens and metallic mirrors, is schematized in Figure 8a, where blue arrows correspond to the excitation LED light source, while green corresponds to the emitted fluorescent light. The metallic mirror reflects the emitted light from the channel surface, induces an optical resonance, and increases the signal by 35–40% [144].

The recorded intensity using this device was ~3.5 times higher than the one obtained on a classical device (Figure 8b). The fluorescent signal increased 8 times, and the signal obtained by 10 µM concentration using the developed device was similar to 100 µM concentration detected in a conventional device without micro-optic elements. In the same context, Shin et al., developed a portable, low-cost, and disposable fluorescent-sensing microfluidic device for on-site detection and quantification of microalgae samples. The chip was equipped with multiple light-emitting diodes (LEDs) for excitation and a silicon photodiode for signal measurements on the same plane. The sensor was able to not only analyze the samples, but also to demonstrate high selectivity by measuring the microalgae concentrations in samples with different levels of turbidity [145]. The fluorescence spectroscopy has also been incorporated into on-chip analysis at high-throughput of a single cell encapsulated in nanoliter aqueous droplets [146] for droplet-based ultra-high-throughput protein screening in directed evolution experiments [147], enzymatic activity [148,149,150], metabolites, and antibodies [151] screening, as well as medical diagnostics [152] and food safety analysis [54].

### 3.3. X-ray Absorption Spectroscopy

In addition to X-ray scattering techniques, X-ray absorption spectroscopy (XAS) is a versatile tool for element-selective detection and speciation characterization [153]. X-ray absorption-measured spectra are characterized by sharp increases in absorption at specific X-ray photon energies (absorption edges), characteristic of the absorbing element, corresponding to the energy required to eject a core electron into the excited electronic states or to the continuum, thus producing a photoelectron. The measurement of transitions from core to excited electronic states is called X-ray absorption near-edge structure (XANES) and gives information regarding the electronic structure (speciation). On the other hand, the absorption measured at energies greater than the threshold for electron release (i.e., the measurement of the transition to the continuum) is called extended X-ray absorption fine structure (EXAFS) and gives information about local structural information around the absorbing element, even from disordered samples, which is an advantage with respect to X-ray crystallography [154]. As a general experimental scheme, the X-rays coming from a source with a wide wavelength spectrum are separated or filtered by a monochromator, where the choice of the probing wavelength is made. A collimator helps align and focus the beam into the sample, which would be the microfluidic device, and the remaining part of the beam that is not absorbed by the analyte is reflected and collected by an X-ray detector.

As one main limitation of the technique, it is worth mentioning that it is unable to distinguish between scattering atoms with little difference in atomic number. However, a more important reason for XAS to not be widely employed in research involving microfluidics is the limited choice of materials for microfluidic fabrication compatible with the technique. The microfluidic devices should be transparent to X-rays, and the polymers and glasses frequently used for microfluidic fabrication may display absorption in the X-ray spectrum that could hamper accurate measurements. Probst et al., integrated XAS measurements to high-throughput droplet-based microfluidics for real-time monitoring of chemical processes. Employing high-frequency time-resolved information XAS experiments on-chip provided access to the early stages of a calcium carbonate precipitation reaction [153]. Analogously, XAS was also employed in a continuous-flow 3D-printed microfluidic reactor to study the formation of palladium nanoparticles [155]. Recently, Ramamoorthy et al., coupled a continuous microreactor platform allowing for ultra-fast reagent mixing (~300 µs) to UV–vis spectrometry, SAXS, and XAS to perform a time-resolved study on the synthesis of ultra-small gold nanoparticles (1–3 nm) in hexane media. The platforms were fabricated in materials 100% compatible with both the chemical system and characterization techniques by using a low-cost procedure, and the formation of nanoparticles could be followed with unprecedented detail from the very first instants of reaction at the sub-millisecond scale, thanks to the fast mixing and the continuous flow and in situ and in operando characterization. UV–vis spectrometry was used to monitor Au(0) concentrations, SAXS to determine the structural evolution of the precursor solution and the formation of the nanoparticles, and XAS for a time-resolved characterization of the evolution of Au speciation [156].

## 4. Scattering-Based Detection Techniques

While optical imaging and spectroscopy-based techniques have been more widespread in microfluidic research, scattering-based techniques are actively evolving and being implemented, particularly in applications related to particle characterization, dynamic processes, and flow-cytometry analysis. This section is divided into two parts addressing, respectively, X-ray and UV–vis scattering techniques. The former is primarily employed for probing the matter at the atomic to the nanometer scale, which permits studying molecular interactions, order, and nanomaterials and small colloids in a wider sense, while the latter is commonly used for the characterization of colloidal particles and biomolecules based on their size distribution.

### 4.1. X-ray Scattering

X-ray scattering techniques are a group of powerful analytical methods in which samples are probed with an incident X-ray beam. The rays are scattered by the sample, and the complex patterns produced by the scattered light are analyzed to investigate the structure of hard and soft matter systems down to the atomic level [157]. This non-destructive method can be applied to various sample types, from crystals to complex biological molecules [158]. X-ray scattering experiments can employ several X-ray sources, from X-ray tubes and rotating anodes to synchrotron radiation. In contrast to synchrotron radiation, conventional X-ray sources, such as X-ray tubes and rotating anodes, have lower brightness, a fixed wavelength, and reduced coherence, which can result in limited time resolution and spatial focusing, impacting their suitability for studies of dynamic processes and anisotropic materials. Equally, when coupling microfluidics to scattering techniques using conventional X-ray sources, due to their low brightness, the choice of fabrication material is crucial to obtain a correct signal-to-noise ratio and preserve the accuracy of the X-ray signals during experiments. Therefore, the materials chosen for the experiments should have low X-ray absorption and minimal interference with the beam to reduce the background noise as much as possible. On the other hand, synchrotron radiation provides high brilliance and brightness, as well as smaller beam sizes, resulting in exceptional spatial and temporal resolution down to nanometers and femtoseconds, respectively. This is also advantageous, as it permits the enhancement of the signal quality, even in high-absorption environments or samples, making it possible to perform measurements that would be difficult or not possible in a laboratory bench. This, together with the possibility of adapting the probing beam size to the microfluidic scale, and the high-frequency time-resolved information are the main reasons why the vast majority of X-ray scattering experiments that are implemented at the microfluidic scale are performed in synchrotron radiation. However, such an intense flux of X-rays can also damage the samples. De facto, the use of microfluidics to perform X-ray scattering studies can be advantageous, as a continuous microfluidic flow can ensure that the sample is exposed to ionizing radiation for short residence times, thus minimizing radiation damage risks [159]. While synchrotrons are of particular interest because of the reasons explained above, they require, in contrast, very high construction and operational and maintenance costs, and they have limited access availability, high operating costs, and, hence, important time constraints for experiments.

Among the main X-ray scattering techniques, small-angle X-ray scattering (SAXS) and wide-angle X-ray scattering (WAXS) are based on the analysis of elastic scattering. SAXS studies the radiation scattered at small angles (0.1–10°), offering nanoscale resolution (1–100 nm) and providing valuable information, including molecular (and nanoparticle) interactions, molecular weights, structural conformations, or folding dynamics for proteins in a solution. In contrast, WAXS focuses on wider angles (>10°), allowing us to reach atomic resolution (0.1–1 nm) and, thus, obtaining information about the structural properties of both organic and inorganic materials. These techniques are often used in combination [157,160,161] to improve the accuracy of structural determination. Their complementary nature is essential when studying complex materials or dynamic processes, offering detailed insights into both large- and small-scale structures for a comprehensive analysis. X-ray diffraction (XRD) is a particular case of X-ray scattering that focuses on scattering angles typically from 5° to 70°. It is principally applied to reveal the inner structure of crystalline materials by examining how incident X-rays interact with the organized atomic arrangement in their crystal lattice, and it is indeed an essential technique in crystallography. Based on the analysis of constructive interference patterns in the scattered beam, it provides highly precise information about lattice spacing and the orientation of crystalline planes.

Review articles on X-ray detection in microfluidics are available in the referenced sources [162,163]. They cover applications of the different scattering techniques in microfluidics for soft matter, life sciences, and structural biology studies up to 2016. Therefore, in this section, we will mainly focus on the most relevant advances in the coupling of these techniques to the micro-scale after this period.

**Figure 9:**
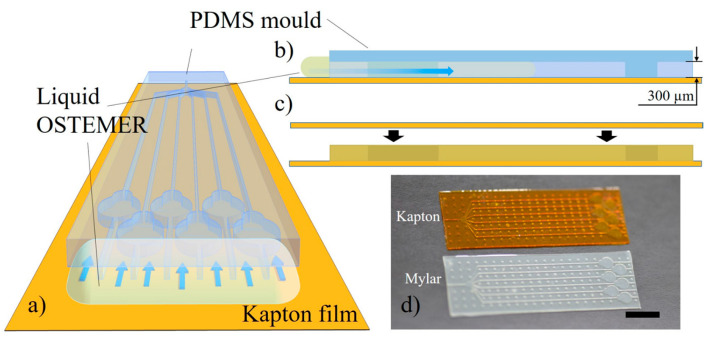
Microchip design and fabrication schematics proposed by Gavira and coworkers for in situ XRD. (**a**,**b**) Operation scheme of a PDMS mold over a Kapton/Mylar film. Liquid OSTEMER formulation fills the gaps between the mold and the Kapton/Mylar film by diffusing by capillary action. (**c**) After UV exposure for OSTEMER cross-linking, the PDMS mold is removed, and the resulting structure is glued to a second Kapton/Mylar film. (**d**). Final view of the X-ray transparent chips, scale bar representing 1 cm. Figure reproduced from [170] with permission from the International Union of Crystallography.

As concerns X-ray diffraction, when working with biological materials and due to the high energy of the X-ray beam, samples generally require cryoprotection to limit beam radiation damage during analysis. One of the main critical factors for applications in structural crystallography is the need for high-throughput screening to search for the optimal crystallization conditions for sample preparation, together with sample handling and manipulation for cryoprotection. On this matter, several microfluidic approaches have been proposed in the literature to tackle this issue, some of them proposed based on data collection at room temperature [164,165,166,167,168,169]. However, the ease of sample preparation–manipulation offered for these devices usually comes at the expense of increased background noise originating from the scattering of chip-fabrication materials, hence limiting the attainable resolution of the diffraction data. Gavira et al., proposed a methodology for low-cost fabrication of X-ray-transparent microchips for in situ crystallization and XRD [170]. The chips (Figure 9) were manufactured using OSTEMER [171] as a main fabrication material for the microchannels, combined with Kapton or Mylar materials to produce sufficiently low scattering background to permit atomic resolution diffraction data collection at room temperature. An unprecedented on-chip atomic resolution for structure determination close to 1.0 Å was demonstrated using lysozyme, thaumatin, and glucose isomerase as model proteins.

**Figure 10:**
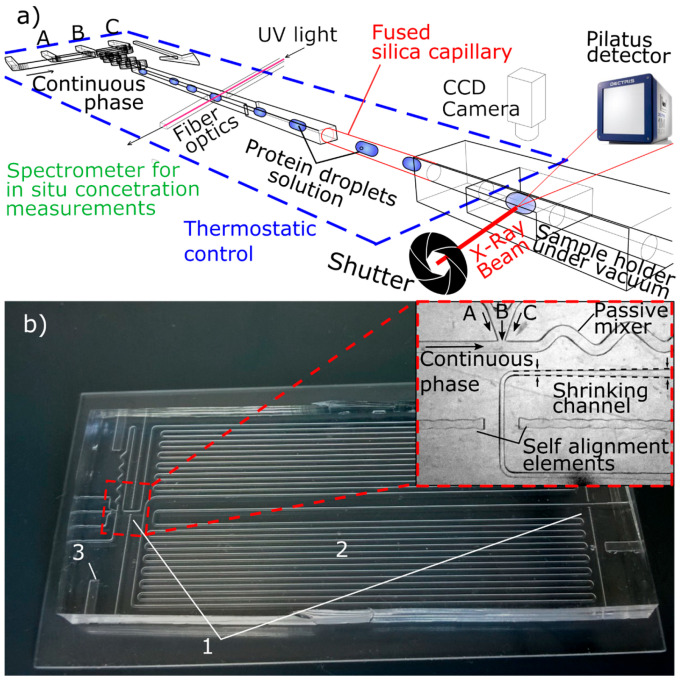
(**a**) Schematics of the droplet-based microfluidic platform proposed by Rodriguez-Ruiz et al., to study protein interactions in solution by combining on-line UV–vis concentration measurements and SAXS [127]. Protein solution droplets at different concentrations are generated and monitored by continuous sensing in the microfluidic platform. Subsequently, they are sent to the SAXS sample holder, where measurements are synchronized with the droplets in movement by actuating in the beam shutter. (**b**) Picture and details of the microfluidic platform showing (1) interrogation areas for photonic detection (detailed in figure inset, where A, B and C inlets are protein, buffer, and precipitant solutions, respectively), (2) serpentine channel for droplet storage, and (3) inlets for temperature probes.

As concerns SAXS/WAXS coupling to the microfluidic scale, recently, Pham and co-workers coupled high-throughput droplet microfluidics made of UV-curable optical adhesive NOA 81 with synchrotron SAXS experiments to study protein crystallization from a solution [172]. Later, Rodriguez-Ruiz and coworkers combined UV–vis spectrometric detection, synchrotron SAXS experiments, and droplet microfluidics for on-chip precise sample preparation, mixing, and real-time protein concentration measurements to probe protein interactions while minimizing radiation damage. The schematics of the analytical platform and setup are presented in Figure 10. The protein gyration radius and structure envelope were calculated as a function of the protein concentration from nanoliter-sized droplets [127].

X-ray scattering experiments have also been widely used in recent years to study the nucleation and growth mechanisms of nanoparticles [156,157,161,173,174], the structural characterization of nano-self-assemblies [175], and the synthesis of other nanomaterials [176]. The small dimensions of microfluidic devices are advantageous for time-resolved X-ray scattering experiments, but it is worth mentioning that experiments can be challenging, not only due to the need for a small beam size but also for the reduced optical paths due to the shallow channels, leading to probing a limited number of scatterers. These issues are mitigated by high-brilliance synchrotron sources, benefiting from their intense flux, as discussed above. Conversely, laboratory beamlines require a higher optimization of X-ray optics and microfluidic chip characteristics to address issues like photon flux or beam size and to minimize chip-related absorption and background scattering. In this context, Levenstein et al., utilized advanced laboratory X-ray instruments for rapid serial scattering and diffraction studies in dilute aqueous solutions, using PMMA, PTFE, and polyimide (Kapton) microfluidic devices, successfully detecting diffraction during a precipitation process with a 25 ms exposure time and obtaining sub-second SAXS patterns of nanoparticles in flowing droplets [177]. In the same way, Rajadewski and coworkers conducted SAXS experiments on a laboratory SAXS bench to study colloidal osmotic compression inside a microfluidic cell using on-chip and in situ fabricated porous membranes. The particular setup for the experiment comprises a microfocus X-ray source, a collimating mirror, and a square scatterless pinhole. The introduction of a mirror and a square scatterless silicon nitride pinhole addressed the challenges faced with decreased photon flux when switching to more divergent optics, significantly increasing the photon flux on the sample up to around $10\times 10^{6}\;ph.s^{-1}$. The microfluidic chip design was chosen to accommodate a relatively large beam size of 250 µm, and the microfluidic cell was built up in OSTEMER material, integrating two 50 µm thick Kapton windows for optimal X-ray transmission. The design maximized the optical path through the sample compared to the chip windows to achieve optimal results. The potential of this setup was demonstrated by probing several well-known colloidal dispersions, such as gold and silica nanoparticles, bovine serum albumin (BSA) macromolecule, and latex nanobeads. The results demonstrated distinct SAXS signatures for gold nanoparticles and BSA macromolecules. Measurable structure factors were also observed for concentrated silica nanoparticle samples. However, a too low signal-to-noise ratio was displayed by the latex nanobeads due to their weak contrast with the solvent, limiting the measurement performances [178].

### 4.2. UV–Vis Scattering Techniques

#### 4.2.1. Forward Visible Light Scattering

Forward light scattering (FSC) relies on analyzing the part of a monochromatic light (frequently a laser beam) scattered in the forward direction, i.e., up to 20° offset from the direction of propagation of the incident wave. The scattered angle can vary depending on the instruments of operation. After interacting with the matter, the scattered light can be collected by a photomultiplier tube (PMT), a photodiode, or a sensor (CCD or CMOS). The latter is an imaging technique usually called digital inline holography and is described in detail in the dedicated following section.

**Figure 11:**
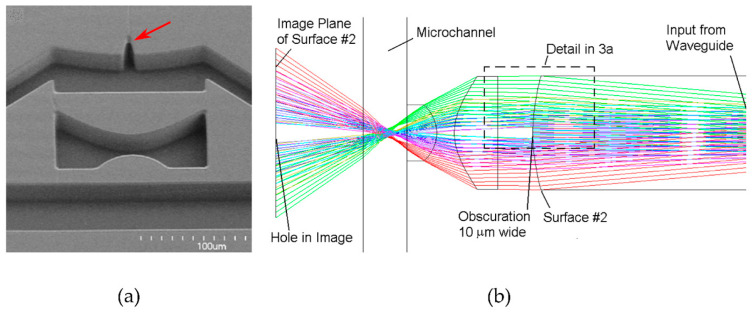
(**a**) Scanning electron microscopy (SEM) image of the lens system incorporating the notch filter. (**b**) Ray-trace simulation of the notch filter in the lens system showing the light gap created in the image plane. Figure adapted from [181], from the journal *Biomedical Optics Express* published by Optica Publishing Group 2013.

Based on this technique, Shivhare and co-workers developed an optofluidic device for mean droplet size measurements and droplet size distributions (DSD) of aqueous droplets in oil by measuring the forward scattered signal (FSC) of the light, detecting sizes as small as 15 µm [179]. Optical fibers, including an excitation fiber orthogonally positioned to the fluidic channel, and a detection fiber set at a 5° angle were integrated to deliver incident light and capture the scattered signal. The droplet sizes were correlated with scattered signals and residence time, the latter corresponding to the time required for the droplet to cross the detection area, which is represented by the pulse width of the detected scattered signal. It was found that the residence times were more relevant to measure the droplet size, as they expressed a linear correlation, unlike the scattered signal. The droplet sizes predicted with this technique matched with the results obtained by microscopy with a difference of 10% for the mean droplet size and a 13% difference for DSD. Lv et al., designed a microfluidic chip incorporating a wave-guided quasi-Bessel beam for detecting scattered signals from cancer cells. Conventional approaches encounter difficulties in accurately capturing the forward small-angle scattered signals of cells, especially at angles lower than 11°. The proposed device involved modulation of the incident Gaussian beam into a quasi-Bessel beam using microprisms and waveguides. The non-diffracting characteristics of the quasi-Bessel beam demonstrated significant improvement (around 50%) in detection accuracy compared to traditional Gaussian lighting methods in microfluidics [180]. Watts et al., introduced a new chip design that not only captures forward scattered light (0.5–5°), enabling beam shaping and utilizing a cost-efficient light source with low quality and high divergence, but also efficiently blocks transmitted light. This design allowed the potential integration of other detection methods and enabled the researchers to create a 10 µm beam geometry and to detect the forward scattered light from 5 µm diameter polystyrene beads. The performance of the device was proven by 0.4% false positive, 6.8% missed events rate, and a coincidence rate of 96.3% determined by simultaneous free-space and on-chip detection schemes. The design included a notch filter in the lens system to deflect the central rays away while leaving the radial rays intact, allowing for an all-planar design for a fully-guided on-chip optical solution with low background noise and higher collection efficiency (Figure 11) [181].

#### 4.2.2. Digital Inline Holography

**Figure 12:**
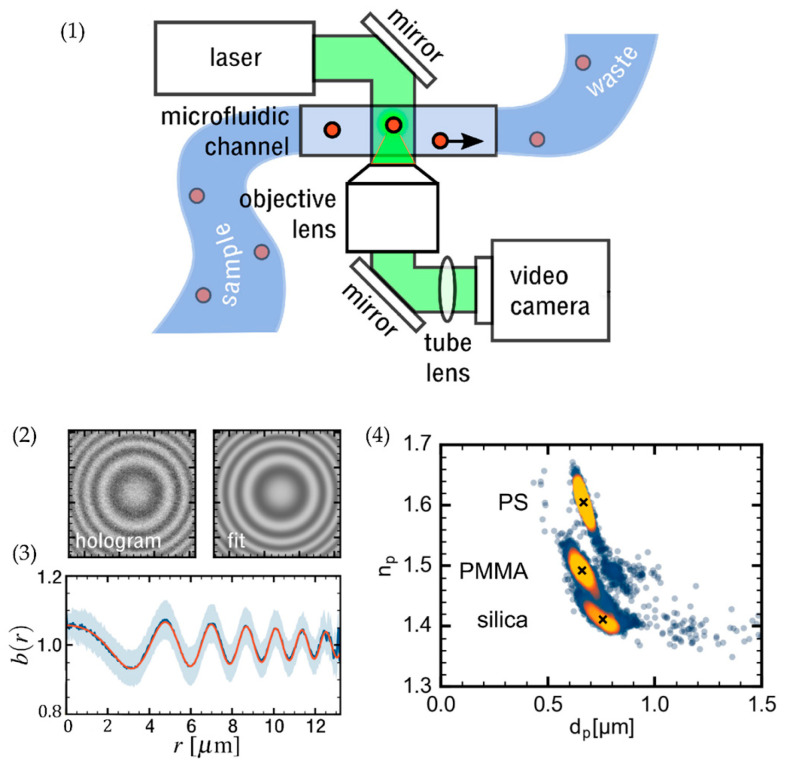
Principle of holographic characterization: (**1**) experimental setup; (**2**) normalized hologram of a polystyrene microbead in water and corresponding fit of the experimental hologram to the prediction of the Lorenz–Mie scattering theory; (**3**) radial profile of the experimental hologram (black) of polystyrene bead dispersed in water overlaid with the fit profile (orange), showing excellent agreement. The blue-shaded region corresponds to the instrumental uncertainty. (**4**) Distribution of three distinct populations of spheres. Figure adapted from [184], from the journal *Water Research* published by Elsevier 2017.

Digital inline holography (DIH), also called lens-free imaging, is an imaging technique based on forward light scattering. The principle of hologram formation can be described by electromagnetic theories such as the Lorenz–Mie scattering theory (LMT) [182], which describes the scattering of plane electromagnetic waves by a particle, usually a homogeneous sphere, or by scalar or vector physical optics theories. The light illuminates an object, and the scattered part of the light combines with the rest of the beam to create an interference pattern. This pattern, known as a hologram, is then recorded by a matrix sensor. This technique can be used to probe the size, the refractive index, and the three-dimensional position and arrangement of individual objects in space. It can simultaneously and quantitatively analyze multiple components of a heterogeneous system (i.e., a mixture including various elements such as cells, droplets, and particles [183,184]), providing results in minutes, and has been used as a guide for the optimization of the on-chip synthesis of polymeric microspheres [185]. DIH is also capable of distinguishing dead and alive yeast cells by the measurement of their refractive index [186], as well as characterizing objects in turbid media [187]. In this regard, Dannhauser et al., characterized the light-scattering profiles (2°–30°) of single polystyrene particles using a CMOS camera-based apparatus. They measured various sizes of polystyrene particles in a flow and compared the obtained scattering profiles to those predicted by the LMT [188]. The setup also allowed simultaneous in-flow measurements of the particle size and refractive index using a microfluidic chip composed of two sections dedicated to particle alignment and particle detection and measurement, respectively. A round glass capillary embedded in the measuring chamber allowed 3D particle alignment along the central axis, thanks to a viscoelastic suspending fluid, making the particles migrate towards the channel’s central axis, as described elsewhere [189]. This kind of flow allowed the laser beam to efficiently interrogate the particles one at a time. Ding et al., developed a lensless holographic imaging portable setup for the characterization of micrometer-sized oil droplets of variable composition, with different lengths of carbon–hydrogen chains (hexane, dodecane, and heptadecane). With the same setup, polystyrene solid particles with four different sizes (10,15, 20, and 30 µm) were analyzed to study the parameters affecting the hologram patterns [190]. They investigated the effects of surfactant addition on the droplet surface properties. The results showed that the addition of surfactant to hexane and heptadecane did not affect the hologram patterns, nor the coating of polystyrene beads by hexane, proving the sensitivity of the technique. The effects of trace amounts of internal components in the oil droplets were also investigated by adding different concentrations of PEA (amino-terminated polyoxypropylene, a fuel additive) to dodecane. They demonstrated that the proposed methodology is sensitive to these trace amounts of additives/impurities, making the portable device interesting for real-time on-site detection [190]. Philips et al., showcased the capabilities of DIH measurements in not only detecting the size and refractive index of individual particles, but also distinguishing between the different types of colloidal particles in a heterogeneous dispersion while individually monitoring their concentration [184]. The concentration of each particle species in a mixture of samples of latex microbeads, oil emulsion droplets, and bacteria were measured using the experimental setup schematized in Figure 12(1). The collimated laser beam illuminated the particle, and the light scattered by a particle was superimposed on the rest of the beam in the focal plane of the microscope. The intensity of the magnified interference pattern was recorded by a video camera. The samples were analyzed, and the size and refractive index were retrieved by comparing the results to the fitted holograms calculated, thanks to LMT (Figure 12(2,3)). This kind of fit could characterize the data for a single particle with instrumental uncertainties of 6 nm for the particle diameter and 2 × 10^−3^ for the refractive index. With such accuracy, the suspension mixture of polymethylmethacrylate (PMMA), polystyrene PS, and silica microspheres can be monitored, and the three particle populations are easily discriminated using a joint distribution of particle diameters and refractive indexes (Figure 12(4)).

Ortiz-Orruno and co-workers applied off-axis holographic nanoparticle tracking analysis—holoNTA—to accurately measure the size and refractive index of materials in heterogeneous nanoparticle suspension. In contrast to the digital inline holographic measurements, the reference and illumination beams took distinct paths. Rather than following the same trajectory, the already scattered beam was subsequently interfered with by the reference beam and recorded by the sensor. The technique combined a high dynamic range with 3D single-particle tracking. This strategy enabled long-term tracking and recording of long trajectories of a single particle by extending the imaging volume, allowing for precise estimates of the scattering amplitude and diffusion coefficient of individual nanoparticles, from which both the refractive index and hydrodynamic diameters were accurately determined (40 to 250 nm) [191]. The approach also enabled digital refocusing, which dramatically increased the volume of observation compared to conventional nanoparticle tracking analysis. Quantitatively, holoNTA extends the depth of focus at least 10 times and prolongs the total observation time for tracked particles, surpassing NTA by at least two orders of magnitude.

#### 4.2.3. Side Light Scattering

Side light scattering, which includes the angles between forward (0–20°) and backward diffraction (180°), is also sensitive to variation in particle size changes and the refractive index. Therefore, side light scattering can be a choice for the applications where such kind of subtle distinctions in these properties are crucial but forward scattering is not achievable. It is less widely used, however, because of the difference in intensity compared to forward diffraction, and the number of works published in the literature is lower. In this regard, Liu and co-workers utilized side scattering detection for detecting and sorting picoliter droplets containing antibiotic-resistant bacteria [192]. Using side scattering at 90°, Pacocha and coworkers quantified bacteria in droplets. Their system was able to distinguish between empty and bacteria-containing droplets at a high frequency of 1.2 kHz. The equivalence of the provided information by scattering detection and fluorescence screening of 12 different bacterial species was demonstrated [193].

#### 4.2.4. Multi-Angle Light Scattering

**Figure 13:**
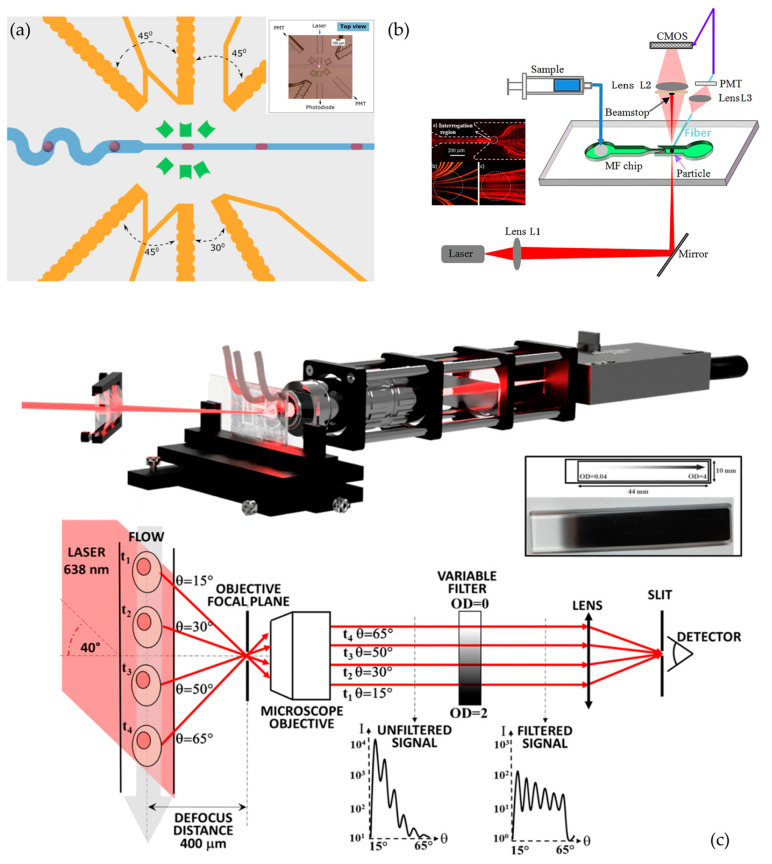
(**a**) The optical interrogation region of the multiparametric optofluidic chip for absorption, fluorescence, and light scattering measurements. Reproduced from [195], from the journal *Biomicrofluidics* published by AIP Publications 2020. (**b**) Schematic diagram of a MALS microscope setup for on-chip measurements. Reprinted from [196], copyright 2017, with permission from Elsevier. (**c**) The µSFC setup: the laser beam is directed at a 40° angle onto the microfluidic channel, positioned 400 µm away from the microscope objective’s focal plane. The objective collects light scattered by particles and focuses it through a lens onto a slit in front of a detector. The virtual image of the slit selects various scattering angles at different positions along particle trajectories. These angles are presented at distinct positions in the back focal plane of the objective, reaching the detector at varying times. To enhance the measurement’s signal-to-noise ratio, a filter with a linearly variable optical density in the back focal plane of the objective reduces the dynamic range. Reproduced from [197], from the journal *Lab on a Chip* published by the Royal Society of Chemistry 2023.

Of course, whenever possible, combining the measurements of forward light scattering and side light scattering is a more powerful approach for the detection, identification, and quantification of nano- and microscale objects. Naturally, this also greatly complicates the coupling with microfluidic devices, as the number of optical accesses required is considerably higher. In this regard, a device for multi-angle light scattering was developed for the characterization of encapsulated cells by Wohlfeil and coworkers [194]. Their study demonstrated the capability to detect microorganisms at the single-cell level in encapsulated environments. Later, the same team miniaturized the detection setup and provided an optofluidic chip with integrated fibers and micro-lenses for light beam focusing and collimation to improve the signal-to-noise ratio. The developed chip was validated by simultaneous measurements of absorbance, fluorescence, and scattered light signals to detect cell density, growth kinetics, and antibiotic inhibition assays in droplets. The incident/excitation and absorption fibers were embedded orthogonally to the fluidic channel. The fluorescence and scattered signal were collected at 45°, 135°, and 225°, respectively, relative to the excitation fiber (see Figure 13a). In addition, the instrument has the potential to insert multiple fibers at the angles noted above for angle-resolved scattering measurements [195]. The multi-angle light scattering technique was also used to analyze waterborne parasites, *E-coli*, and impurities in water. The proposed setup consists of a microfluidic chip in which targeted particles pass through a focused 200 µm laser beam emitted by a fiber-pigtailed laser diode. A focusing lens images the forward scattered light onto a CMOS camera. A beam stop, located along the scattered light path, blocks incident and bright zero-order scattered light to protect the camera sensor from saturation. The focusing lens collects scattered light in the range of 8° to 38°. A fiber embedded in an etched groove perpendicular to the flowing channel collects focused side-scattered light onto a photomultiplier tube (PMT) detector, which converts the light pulse into a voltage signal, amplified with adjustable gain to increase the sensitivity (see Figure 13b). The setup was first tested with polystyrene microspheres, and the size and relative refractive indices for the particles were derived with a respective accuracy of 60 nm for particle size and 0.002 for refractive index by comparing the experimental scattering patterns with the theoretical ones. Subsequently, utilizing a classification machine learning algorithm (support-vector-machine), up to 3000 waterborne parasites could be identified within one minute, with a mean accuracy higher than 96% [196]. Recently, Reale et al., developed a microfluidic scanning flow cytometer (µSFC) that achieved on-chip angle-resolved light scattering measurements for high-throughput determination of the size and refractive index of single cells. A single photoreceiver was used for the measurements, together with a low-cost linearly variable density (OD) filter (Figure 13c). The purpose of using this filter is to reduce the dynamic range of the signal, optimize the use of the photodetector range, and simultaneously increase its signal-to-noise ratio. The µSFC achieves superior performance over the gold-standard flow cytometry and fluorescence-activated cell sorter machines for particle size estimation and label-free analysis. The µSFC also validated the feasibility of analyzing biological samples by studying monocytes in a blood cell sample, yielding values consistent with the literature. The proposed system demonstrated great potential for integration within other lab-on-a-chip systems for multiparametric cell analysis and next-generation point-of-care diagnostics applications [197].

In this setup, a laser beam focused by a cylindrical lens is scattered by the particles flowing in the microchannel. Scattered light is collected by a microscope objective and focused onto a photoreceiver sensor by a lens (Figure 13c). The microchannel is positioned in the out-of-focus plane 400 µm from the objective focal plane. A slit placed on the lens focal plane is used to selectively collect the different scattered angles at different times. The angular resolution depends on the out-of-focus distance and on the slit width, where an increased defocus distance improved the angular resolution but decreased the signal intensity. The objective numerical aperture determined the collected angular range, and the excitation laser beam angle relative to the microchannel shifted the center of the measured angle.

#### 4.2.5. Dynamic Light Scattering (DLS)

Dynamic light scattering (DLS), also known as photon correlation spectroscopy, is commonly applied for the investigation of nanoparticle size distribution (PSD) in a solution, and it is frequently used in laboratories. In colloidal solutions, particles undergo the Brownian motion, and factors such as particle size, thermal energy, viscosity, and morphology influence their average motion velocity. In typical DLS measurement, a coherent light source (typically a laser beam) is used, and it is scattered from the particles in suspension. Since the particles are in constant motion, the changing intensity of this pattern is used to calculate an autocorrelation function (ACF). Analyzing the ACF yields the velocity distribution, more precisely, the diffusion coefficient of particles and, subsequently, their hydrodynamic size distribution, utilizing the Stokes–Einstein equation. It is worth noting that the hydrodynamic radius ($R_{H}$) of the particles can vary in different conditions (pH values, temperatures, surfactants, and type of solvent) and does not systematically match the geometrical size [198]. DLS is applicable for particles ranging from several nanometers to a few microns. However, DLS measurements become challenging in the presence of any motion (flow, thermic gradient, etc.), as the impact of Brownian motion becomes negligible compared to the displacement induced by the other physical effects. Therefore, DLS appears hard to implement on microfluidic devices, and interpretation of the results is challenging due to the additional shear and flow contributions to the intensity fluctuations and autocorrelation functions [199]. However, Chastek et al., integrated DLS into a microfluidic device using fiber optic probes in direct contact with the sample solution [200]. A year later, the same group introduced a microfluidic platform integrating DLS measurements to study the synthesis of block copolymer micelles. Large detection areas of 790 µm × 790 µm were conceived and total flow rates as low as 500 µL/h were used to implement the technique on-chip. The system was capable of identifying the size and aggregation behavior of micelles ($D_{H}\sim 25\;nm$) [201]. Destremaut et al., conducted on-line dynamic light scattering measurements in a Poiseuille microfluidic flow by estimating shear rates suitable for size distribution measurements. The developed fiber-optics-integrated microfluidic system was adapted for continuous DLS measurements, utilizing continuous viscosity monitoring of a two-fluid mixture. This approach allowed estimation of the flow rate ranges suitable for DLS size measurements in pressure-driven flow, which were experimentally confirmed using monodisperse calibrated nanoparticles [202]. Chen et al., introduced a dual-angle fiber (30° and 45°) DLS system integrated into a microfluidic chip for precise polystyrene nano- and submicron particle size distribution measurements. This system demonstrated reproducibility, maintaining precision even at high concentrations (14 mg/mL), and reduced the influence of multiple scattering on high concentration sample measurements by consuming minimal sample volume (30 µL) [203]. A year later, the same team measured standard calibrated particles of 80 to 800 nm, with a detection error in size measurements less than 7%. Overall, the developed instrument allowed two-angle DLS measurements, eased the optical alignment, reduced multiple scattering, and allowed high-throughput measurements [204]. Torquato et al., proposed different integrated fiber-optic DLS systems, utilizing both a capillary-connected microdevice and direct measurements on a glass chip. In this study, the flow-DLS systems were able to measure the size and polydispersity of model particles, including micellar and dilute polymer solutions, and colloidal dispersions of different polystyrene and silica nanoparticles at different velocities (0–16 cm/s). Additionally, the composition characterization of a two-component micellar solution was performed, and it was found that the micelle hydrodynamic radius increased with the NaCl concentration from ~0.7 to 2 nm. The system was capable of carrying out composition mapping measurements under flow with dynamically varying compositions, with integration times ranging from approximately 3 to 10 s. It was found that a glass capillary-connected device featuring a 1 mm internal diameter has certain advantages in terms of experimental robustness and ease of operation. Reducing the channel dimensions increases the shear gradients and requires high precision in optical alignment, and issues such as parasitic light refraction and reflections from the channel walls become more prominent [205].

## 5. Conclusions

Over the past decade, the field of microfluidics has witnessed significant advancements in in situ photonic detection implementation, revolutionizing the way researchers manipulate and analyze fluids and particles at the microscale. One notable trend involves the integration of photonic technologies to enhance sensitivity, resolution, and real-time monitoring capabilities in microfluidic systems. Miniaturization of photonic devices has been a key focus, enabling the creation of compact and efficient sensors for various applications within microfluidics. These innovations have empowered researchers to explore new frontiers in chemical and biological analyses with unprecedented precision. Additionally, the integration of on-chip photonic components, such as waveguides, mirrors, lenses, and resonators, has facilitated the creation of integrated microfluidic platforms. This convergence of technologies has enabled seamless coupling between optics and fluidics, paving the way for more robust and versatile systems, and allowing integration of increasingly complex photonics techniques.

In this review, we have thoroughly introduced the most relevant and recent developments in a wide range of on-chip photonic detection techniques, covering UV–vis, near-infrared, terahertz, and X-ray-based detection techniques for different characterizations, ranging from punctual spectroscopic or scattering-based measurements to different types of mapping/imaging. The principles of the techniques and their interest have been discussed through their application to different systems. As a main conclusion, a summary of the discussed articles, grouped by detection technique with their respective advantages and limitations, and a mention of their on-chip implementation and integration feasibility is proposed in Table 2.

In the future, the integration of AI into these devices heralds a transformative change in signal processing, improving sensitivity and real-time analysis through machine learning. The automation of experimental optimization by AI will streamline processes and facilitate signal processing, guaranteeing the efficiency and reproducibility of measurements and experiments. This synergy should lead to significant breakthroughs in the use of these techniques.

## Figures and Tables

**Table 1:** Partial picture of the literature available on the Web of Science Core Collection, regarding three main applications: chemistry, materials science, and biology.

Keywords and Booleans	Total Number of Papers	Number of Papers in Chemistry	Number of Papers in Material Science	Number of Papers in Biological Science
(SAXS OR X-ray scattering) AND (microfluidics OR Lab-on-Chip)	69	61	30	14
(UV-vis scattering) AND (microfluidics OR Lab-on-Chip)	12	8	8	-
(Optical imaging) AND (microfluidics OR Lab-on-Chip)	842	257	223	91
(spectroscopic OR spectrophotometric)) AND (microfluidics OR Lab-on-Chip)	248	149	27	37
Total number of papers per application		475	288	142

**Table 2:** Summary of the articles discussed in this work, including the advantages and disadvantages of the presented on-chip photonic detection techniques, together with a mention of their on-chip implementation/integration feasibility (“+” standing for simple, “−” standing for complex, and “×” standing for not possible).

Category	Detection Technique	Advantages	On-Chip Implementation/Integration	Drawbacks
Optical imaging	Bright-field Imaging [37,38,39,40,41,42,43,44,45,46,47,48,49,50]	Simple and cost-effective Real-time observation High-throughput	+/×	Low contrast of transparent objects Limited spatial resolution Does not provide 3D information
	Epifluorescence Microscopy [56,57,58,59,60,61]	Highly sensitive Fast acquisition Real-time imaging	+/×	Photobleaching/phototoxicity in continuous exposure Requires labeling or derivation Background noise from autofluorescence in plastic chip materials Low axial resolution Limited depth of field
	Confocal Microscopy [62,63,64]	Highly sensitive Enhanced axial resolution for 3D reconstruction	+/×	Slower acquisition times Photobleaching/phototoxicity in continuous exposure Requires labeling or derivation Background noise from autofluorescence in plastic chip materials
	Light-sheet Microscopy [65,66,67,68]	Highly sensitive Selective specimen illumination Reduced photobleaching and phototoxicity Valuable for dynamic studies, high-contrast imaging	−/−	Requires labeling or derivation Background noise from autofluorescence in plastic chip materials
Spectroscopy-based detection	Raman Spectroscopy [72,73,74,75,76,77,78,81,82,83,84,85,86,87,88,89,90,91,92,93,94,95,96]	Label-free Non-invasive Online Rapid analysis Unique fingerprints for chemical identification	+/+	Limited sensitivity for low concentration samples Background signals from microfluidic chips
	FTIR Spectroscopy [99,100,101,102,103,104,105,106]	Label-free Non-invasive Provides information on composition and structure and molecular interaction Time-resolved mapping	+/−	Limited application for aqueous samples due to the absorption of IR spectra
	Terahertz Spectroscopy [108,110,111,112,113,114,115,116,117]	Low-energy makes it suitable for biological samples Real-time measurements High spatio-temporal resolution	−/×	Requires careful material selection to minimize THz absorption, high absorption of aqueous samples
	Absorption Spectrometry [120,121,122,123,127,128,129,130]	Label-free, Online analysis Real-time quantitative analysis	+/+	Challenges in achieving low limit of detection in microfluidics due to the small optical path lengths
	Light Extinction Spectroscopy [131,132,133,136]	Label-free Online Real-time quantitative analysis Characterization of colloidal particle distribution in stationary and dynamic suspensions	+/+	Limited to optically dilute colloidal suspensions Challenges in achieving low limit of detection in microfluidics due to small optical path lengths
	Photothermal Spectroscopy [138,139,140,141,142]	Label-free Highly sensitive Rapid quantitative measurements	−/×	High cost of required experimental equipement
	Fluorescence Spectroscopy [54,143,144,145,146,147,148,149,150,151,152]	Highly sensitive Fast response time High throughput Real time monitoring	+/+	Photobleaching/phototoxicity in continuous exposure Requires labeling or derivation Background noise from autofluorescence in plastic chip materials
	X-ray Absorption Spectroscopy [153,155,156]	Element selective characterization of samples High throughput Time-resolved experiments allow monitoring of early stages of reaction Reveals crystal structures of samples	−/×	Polymers and glass might display absorption in X-ray interfering accurate measurements Often requires synchrotron radiation
Scattering-based detection	X-ray Scattering [157,158,159,161,162,163,164,165,166,167,168,169,172,173,174,175,176]	Provides insights of atomic level structures Applicable to wide range of samples	−/×	Material selection for accurate measurements Often requires synchrotron radiation
	Forward Visible Light Scattering [179,180,181]	Sensitive to particles size and refractive index measurements	−/−	Limited structural information May face challenges when interpreting the scattered patterns when dealing with non-spherical particles
	Digital Inline Holography [183,184,185,186,187,188,190,191]	Provides 3D information of spherical particles Allows accurate size and refractive index measurements	+/−	The surface roughness of the microfluidics channels cause high noise levels May face challenges when interpreting the scattered patterns when dealing with non-spherical particles
	Side Light Scattering [192,193]	Sensitive to variations in particle size and refractive index	−/−	May face challenges when interpreting the scattered patterns when dealing with non-spherical particles
	Multi-angle Light Scattering [194,196,197]	Provides information on shape, size, and granularity of the samples	−/−	May face challenges when interpreting the scattered patterns when dealing with non-spherical particles
	Dynamic Light Scattering [198,199,201,202,203,204,205]	Measures colloidal particle size and polydispersity	−/−	Challenging in-flow measurements Parasitic light refraction and reflections from channel walls
